# Catalyzing Transcriptomics Research in Cardiovascular Disease: The CardioRNA COST Action CA17129

**DOI:** 10.3390/ncrna5020031

**Published:** 2019-03-29

**Authors:** Clarissa Pedrosa da Costa Gomes, Bence Ágg, Andrejaana Andova, Serdal Arslan, Andrew Baker, Monika Barteková, Dimitris Beis, Fay Betsou, Stephanie Bezzina Wettinger, Branko Bugarski, Gianluigi Condorelli, Gustavo José Justo da Silva, Sabrina Danilin, David de Gonzalo-Calvo, Alfonso Buil, Maria Carmo-Fonseca, Francisco J. Enguita, Kyriacos Felekkis, Peter Ferdinandy, Mariann Gyöngyösi, Matthias Hackl, Kanita Karaduzovic-Hadziabdic, Jan Hellemans, Stephane Heymans, Markéta Hlavackova, Morten Andre Hoydal, Aleksandra Jankovic, Amela Jusic, Dimitris Kardassis, Risto Kerkelä, Gabriela M. Kuster, Päivi Lakkisto, Przemyslaw Leszek, Mitja Lustrek, Lars Maegdefessel, Fabio Martelli, Susana Novella, Timothy O’Brien, Christos Papaneophytou, Thierry Pedrazzini, Florence Pinet, Octavian Popescu, Ines Potočnjak, Emma Robinson, Shlomo Sasson, Markus Scholz, Maya Simionescu, Monika Stoll, Zoltan V. Varga, Manlio Vinciguerra, Angela Xuereb, Mehmet Birhan Yilmaz, Costanza Emanueli, Yvan Devaux

**Affiliations:** 1 Cardiovascular Research Unit, Luxembourg Institute of Health, L-1445 Strassen, Luxembourg; Clarissa.PedrosaDaCostaGomes@lih.lu; 2 Department of Pharmacology and Pharmacotherapy, Semmelweis University, 1085 Budapest, Hungary; agg.bence@med.semmelweis-univ.hu (B.Á.); peter.ferdinandy@pharmahungary.com (P.F.); varga.zoltan@med.semmelweis-univ.hu (Z.V.V.); 3 Department of Intelligent Systems, Jozef Stefan Institute, 1000e Ljubljana, Slovenia; andrejaana.andova@ijs.si (A.A.); mitja.lustrek@ijs.si (M.L.); 4 Department of Medical Biology, Faculty of Medicine, Cumhuriyet University, 58140 Sivas, Turkey; arserdal@yahoo.com; 5 Centre for Cardiovascular Science, The Queen’s Medical Research Institute, University of Edinburgh, Edinburgh EH16 4TJ, UK; Andy.Baker@ed.ac.uk; 6 Institute for Heart Research, Centre of Experimental Medicine, Slovak Academy of Sciences, 84104 Bratislava, Slovakia; monika.bartekova@savba.sk; 7 Biomedical Research Foundation, Academy of Athens, 11527 Athens, Greece; dbeis@bioacademy.gr; 8 Integrated BioBank of Luxembourg, L-3550 Dudelange, Luxembourg; Fay.Betsou@ibbl.lu; 9 Faculty of Health Sciences, University of Malta, MSD 2080 Msida, Malta; stephanie.bezzina-wettinger@um.edu.mt (S.B.W.); angela.a.xuereb@um.edu.mt (A.X.); 10 Faculty of Technology and Metallurgy, University of Belgrade, 11000 Belgrade, Serbia; branko@tmf.bg.ac.rs; 11 Department Cardiovascular, Humanitas Research Hospital, 20089 Rozzano, Italy; gcondorelli@yahoo.com; 12 Institute for Experimental Medical Research, Oslo University Hospital and University of Oslo, PB 4956 Nydalen, NO-0424 Oslo, Norway; g.j.j.d.silva@medisin.uio.no; 13 Firalis, 68330 Huningue, France; sabrina.danilin@firalis.com; 14 Institute of Biomedical Research of Barcelona (IIBB)—Spanish National Research Council (CSIC), 08036 Barcelona, Spain; david.degonzalo@gmail.com; 15 Mental Health Center Sct. Hans, 4000 Roskilde, Denmark; alfonso.buil.demur@regionh.dk; 16 Instituto de Medicina Molecular, Faculty Medicine, University Lisboa, 1649-028 Lisboa, Portugal; carmo.fonseca@medicina.ulisboa.pt (M.C.-F.); fenguita@medicina.ulisboa.pt (F.J.E.); 17 Department of Life and Health Sciences, School of Sciences and Engineering, University of Nicosia, 2417 Nicosia, Cyprus; felekkis.k@unic.ac.cy (K.F.); papaneophytou.c@unic.ac.cy (C.P.); 18 Department of Cardiology, Medical University of Vienna, 1090 Vienna, Austria; mariann.gyongyosi@meduniwien.ac.at; 19 TAmiRNA, 1190 Vienna, Austria; matthias.hackl@tamirna.com; 20 Faculty of Engineering and Natural Sciences, International University of Sarajevo, 71210 Sarajevo, Bosnia and Herzegovina; kanita@ius.edu.ba; 21 Biogazelle, 9052 Zwijnaarde, Belgium; jan.hellemans@biogazelle.com; 22 School for Cardiovascular Diseases, Faculty Health, Medicine and Life Sciences, Maastricht University, 6229ER Maastricht, The Netherlands; s.heymans@maastrichtuniversity.nl; 23 Department of Developmental Cardiology, Institute of Physiology of the Czech Academy of Sciences, 142 20 Prague, Czech Republic; Marketa.Hlavackova@fgu.cas.cz; 24 Department of Circulation and Medical Imaging, Faculty of Medicine and Health Sciences, NTNU Norwegian University of Technology and Science, NO-7489 Trondheim, Norway; morten.hoydal@ntnu.no; 25 Department of Physiology, Institute for Biological Research “Siniša Stanković,” University of Belgrade, 11000 Belgrade, Serbia; aleksandra.jankovic@ibiss.bg.ac.rs; 26 Department of Biology, Faculty of Natural Sciences and Mathematics, University of Tuzla, 75000 Tuzla, Bosnia and Herzegovina; amela.jusic@untz.ba; 27 Department of Basic Sciences, University of Crete Medical School and Institute of Molecular Biology and Biotechnology (IMBB), Foundation for Research and Technology of Hellas, Heraklion 71003, Crete, Greece; kardasis@imbb.forth.gr; 28 Research Unit of Biomedicine, University of Oulu, 90014 Oulu, Finland; Risto.Kerkela@oulu.fi; 29 Department of Biomedicine and Clinic of Cardiology, University Hospital and University of Basel, 4031 Basel, Switzerland; Gabriela.Kuster@usb.ch; 30 Minerva Institute for Medical Research, 00290 Helsinki, Finland; paivi.lakkisto@helsinki.fi; 31 The Cardinal Stefan Wyszynski Institute of Cardiology, 04-628 Warsaw, Poland; przemyslaw.leszek@ikard.pl; 32 Center for Molecular Medicine, Karolinska Institute, 17176 Stockholm, Sweden; lars.maegdefessel@ki.se; 33 Molecular Cardiology Laboratory, IRCCS-Policlinico San Donato, 20097 San Donato Milanese, Milan, Italy; Fabio.Martelli@grupposandonato.it; 34 Department of Physiology, University of Valencia and INCLIVA Biomedical Research Institute INCLIVA, 46010 Valencia, Spain; susana.novella@uv.es; 35 Regenerative Medicine Institute REMEDI, National University of Ireland Galway (NUIG), H91 TK33 Galway, Ireland; Timothy.obrien@nuigalway.ie; 36 Experimental Cardiology Unit, Division of Cardiology, Department of Cardiovascular Medicine, University of Lausanne Medical School, 1011 Lausanne, Switzerland; thierry.pedrazzini@chuv.ch; 37 INSERM, Institut Pasteur de Lille, 59019 Lille Cedex, France; florence.pinet@pasteur-lille.fr; 38 Babes-Bolyai University, 400084 Cluj-Napoca, Romania; opopescu.ubbcluj@gmail.com; 39 University Hospital Centre Sisters of Charity, Institute for clinical medical research and education, 10 000 Zagreb, Croatia; ines.potocnjak@yahoo.com; 40 Department of Cardiology, Centre for Heart Failure Research, Faculty of Health, Medicine and Life Sciences, Maastricht University, 6229 ER Maastricht, The Netherlands; e.robinson@maastrichtuniversity.nl; 41 Institute for Drug Research, Faculty of Medicine, The Hebrew University, 91120 Jerusalem, Israel; Shlomo.sasson@mail.huji.ac.il; 42 Institute for Medical Informatics, Statistics and Epidemiology IMISE, Medical Faculty, University of Leipzig, 04107 Leipzig, Germany; Markus.Scholz@imise.uni-leipzig.de; 43 Institute of Cellular Biology and Pathology “Nicolae Simionescu”, 050568 Bucharest, Romania; Maya.Simionescu@icbp.ro; 44 Westfälische Wilhelms-Universität Münster, Institute of Human Genetics, Department of Genetic Epidemiology, 48149 Münster, Germany; mstoll@uni-muenster.de; 45 International Clinical Research Center, 656 91 Brno, Czech Republic; manlio.vinciguerra@fnusa.cz; 46 Dokuz Eylul University, Faculty of Medicine, Department of Cardiology, 35340 Izmir, Turkey; prof.dr.mbyilmaz@gmail.com; 47 National Heart & Lung Institute, Faculty of Medicine, Imperial College London, London W12 0HH, UK; c.emanueli@imperial.ac.uk

**Keywords:** cardiovascular disease, transcriptomics, best practices and guidelines, translational research, personalized medicine

## Abstract

Cardiovascular disease (CVD) remains the leading cause of death worldwide and, despite continuous advances, better diagnostic and prognostic tools, as well as therapy, are needed. The human transcriptome, which is the set of all RNA produced in a cell, is much more complex than previously thought and the lack of dialogue between researchers and industrials and consensus on guidelines to generate data make it harder to compare and reproduce results. This European Cooperation in Science and Technology (COST) Action aims to accelerate the understanding of transcriptomics in CVD and further the translation of experimental data into usable applications to improve personalized medicine in this field by creating an interdisciplinary network. It aims to provide opportunities for collaboration between stakeholders from complementary backgrounds, allowing the functions of different RNAs and their interactions to be more rapidly deciphered in the cardiovascular context for translation into the clinic, thus fostering personalized medicine and meeting a current public health challenge. Thus, this Action will advance studies on cardiovascular transcriptomics, generate innovative projects, and consolidate the leadership of European research groups in the field. COST (European Cooperation in Science and Technology) is a funding organization for research and innovation networks (www.cost.eu).

## 1. Introduction

Cardiovascular diseases (CVD) remain the main cause of death worldwide despite technological and medical advances and is responsible for debilitating a significant number of patients annually. To discover novel diagnostic and prognostic tools and develop new treatments, understanding the molecular mechanisms in a comprehensive view is essential. Such knowledge can forward personalized medicine, where interventions are tailored to the individual patient, potentially improving the lives of millions of people, and reducing healthcare costs.

One way to promote this area is by studying the transcriptome, which is the set of all different types of RNA produced in a cell and that may be involved in the pathogenesis of several diseases, including cardiovascular ones. However, the human transcriptome is much more complex and challenging to study than previously thought, and new types of RNA are frequently revealed. Currently, there are relatively isolated research groups working on the same subject and sometimes using different standards to generate data, making it harder to compare and reproduce results. This is particularly true when aiming to develop products for personalized medicine because they must deliver accurate information and high reproducibility.

This European Cooperation in Science and Technology (COST) Action will provide a platform to coordinate the efforts of different groups and offer opportunities for collaboration between clinicians and scientists from interdisciplinary backgrounds to more rapidly decipher the role of different forms of RNA in CVD and allow for the transfer of such knowledge into practical applications for diagnostics and therapies. CardioRNA members aim to refine and implement guidelines for transcriptomic studies in CVD, tackling the whole investigation pipeline to improve comparison between studies from different laboratories and reproducibility of results, allowing faster development of new RNA-based tools for personalized medicine. This Action also aims to enable enhancement and continuity of the network efforts in improving the understanding of the transcriptome in the cardiovascular context and advancement of personalized medicine. Finally, it has the goal to provide a platform to train the next generation of researchers and students in the field.

## 2. Relevance and Timeliness

Non-communicable diseases are the major global disease burden, with CVD as the leading cause of death, accounting for 17.5 million deaths per year, a number that is estimated to increase [1]. The socioeconomic burden of CVD due to premature deaths and reduced quality of life is substantial. It has been estimated to cost €210 billion annually to the European Union alone [2]. Considerable parts of the world are at significant risk for CVD, which, as a chronic disease, may be caused by cumulative biological, behavioral, and social risks. Nonetheless, the main risk factors contributing to CVD tend to be consistent worldwide: Hypertension, diabetes, poor diet and obesity, physical inactivity, and smoking [3].

The modern lifestyle that promotes sedentary behavior and ingestion of high-calorie foods has resulted in an obesity epidemic around the globe and is related to the increasing incidence of chronic diseases, particularly cardiovascular ones [4]. Such aspects of modern lifestyle result in abnormal gene expression, contributing to the development of pathologies that significantly increase the risk for CVD development, such as obesity, atherosclerosis, type 2 diabetes, and hypertension. The high and increasing prevalence of these chronic conditions has contributed to the pandemic of CVD [5].

**Figure 1:**
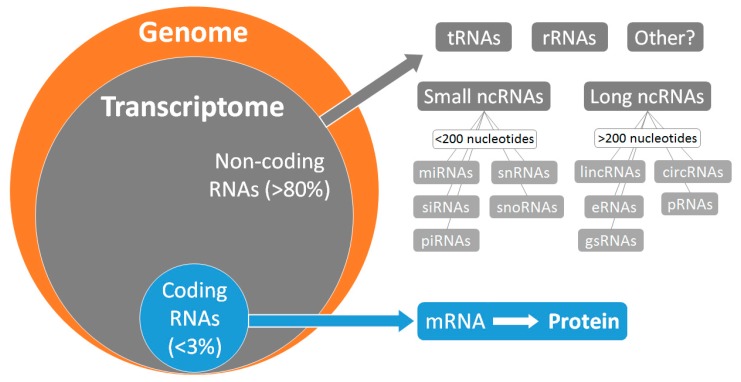
Coding and non-coding RNAs in the human genome. tRNAs, transfert RNAs; rRNAs, ribosomal RNAs; ncRNAs, noncoding RNAs; miRNAs, microRNAs; siRNAs, small-interfering RNAs; snRNAs, small nuclear RNAs; snoRNAs, small nucleolar RNAs; piRNAs, piwi-interacting RNAs; linRNAs, long intergenic RNAs; eRNAs, extracellular RNAs; gsRNAs, germline small RNAs; circRNAs, circular RNAs; pRNAs, promoter-associated RNAs; mRNA, messenger RNA.

Environmental factors, such as those mentioned above, can affect gene expression, which is reflected by the transcriptome. For many years, there was an emphasis on the study of protein-coding genes giving rise to messenger RNA (mRNA). More recently, the emergence of non-coding RNA genes (ncRNA) has allowed the scientific community to appreciate the full complexity of the transcriptome. The discovery of various new classes of RNAs and their different functions has had profound implications for molecular biology and medical research (Figure 1). The importance of ncRNAs in physiological and pathological processes, such as CVD, has been frequently reinforced, and they have major regulatory roles in gene expression [6,7]. Thus, understanding the interactions between the different types of RNA, both coding and non-coding, and how they affect gene expression in CVD, is necessary to forward CVD management.

In addition, knowledge of the transcriptome regulation and recent technological developments to manipulate gene expression (e.g., RNA interference, antagomiRs, gapmers, short hairpin RNA viral-mediated delivery of ncRNAs) allow investigation of the role and value of RNAs to personalize healthcare of patients affected by CVD. Thus, the transcriptome, and particularly ncRNAs, may have a more direct application in the clinical practice. Importantly, although most ncRNAs are found inside cells, they have been consistently identified in body fluids, including the blood [8]. The types and amounts of different ncRNAs vary and have distinct and specific profiles in different pathophysiological states [9,10], leading to the possibility of using these molecules as non-invasive markers of disease [11,12]. Several studies have suggested that specific ncRNA profiles may be used as diagnostic and prognostic tools for different pathologies, including cardiovascular ones [13].

Early and accurate diagnosis and prognosis for CVD patients can optimize clinical decision making to better adjust interventions for individual patients. Currently, optimal treatment selection and dosage are restricted due to limited awareness of molecular and environmental information of patients [14]. A shift in medicine to focus on individuals rather than populations promises a more proactive and predictive approach [15]. This is the concept of personalized medicine, which is committed to inspect, diagnose, and monitor risk to ensure that patients receive treatments tailored to their molecular and individual configuration [14]; or, if not to the individual, treatments tailored to a subgroup of individuals that share similar traits and molecular outline [16].

However, personalized medicine presently represents a more theoretical concept than a practical one, with the one-size-fits-all approach being still broadly used to manage CVD. To put the promise of personalized medicine in practice, tools that stratify patients and improve healthcare need to be accessible in the clinic, making translational research essential, meaning that basic research findings are converted into usable applications for clinicians and patients. The basis of personalized medicine includes targeted therapies and biomarkers, which are molecular indicators of diagnostic and prognostic disease and treatment efficacy. The transcriptome provides an important source of therapeutic agents and biomarkers as continually demonstrated by different research [17,18,19]. Although new technologies to study the transcriptome enable information of its role in CVD mechanisms, much remains to be elucidated as new levels of complexity are unveiled. The slow speed at which advances are occurring in translational research suggests there are bottlenecks to be resolved [15]. Thus, there is a crucial and urgent need for a collective effort to advance in the field by deciphering the role of the transcriptome in CVD and create new possibilities for the development of innovative products for CVD management. 

## 3. Objectives

**Figure 2:**
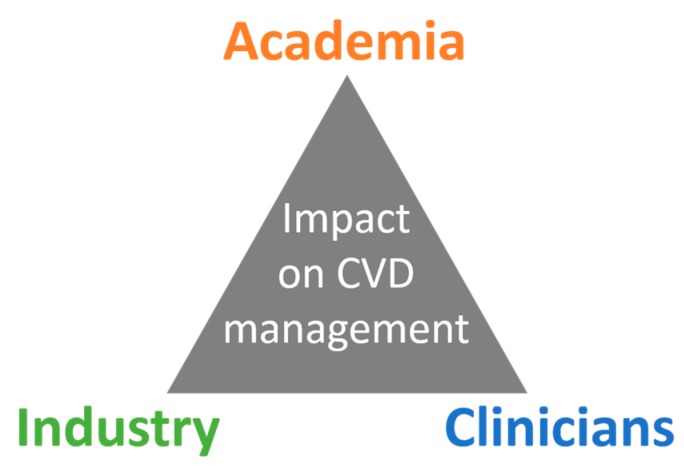
Different backgrounds of the network members and their interactions to impact cardiovascular diseases (CVD) management, thus healthcare.

This COST Action aims to accelerate the understanding of transcriptomics in CVD so personalized medicine advances faster in this field, addressing a current public health challenge. It proposes to constitute a network to offer opportunities for collaboration between clinicians, academic researchers from interdisciplinary backgrounds, and industry to achieve breakthroughs and allow the transfer of basic science into usable applications (Figure 2). CardioRNA aims to refine guidelines from the design to the analysis of transcriptomics data to enable better comparison between studies from different laboratories and reproducibility of results, facilitating the development of products.

The Action will lead to creating new possibilities of basic research and product development for personalized medicine. CardioRNA members believe that by coordinating research activities through an interdisciplinary network, more meaningful results can be achieved. Furthermore, the platform that will be provided by the Action will allow easier public–private partnerships to support the translation of the new knowledge to the clinic.

Researchers from pertinent disciplines will connect through CardioRNA to organize activities to achieve the research objectives (Table 1).

Additionally, CardioRNA will convene researchers from COST countries and International Partner Countries (IPC) to increase scientific dialogue and provide training in the role of transcriptomics in CVD through different types of meetings, involving students and early career investigators (ECI). It will support them to attend meetings, training schools, and short-term scientific meetings (STSM) within the Action.

## 4. Progress Beyond the State-of-the-Art and Innovation Potential

The transcriptome represents all genes expressed in a tissue in a given biological state. In contrast to DNA sequence that is constant in an individual, there is great variation in gene expression in different tissues and different pathophysiological circumstances [20]. Specific signatures of the transcriptome have been associated with CVD phenotypes and prognosis, while the investigation of RNA frequently points to the structural and functional versatility of these molecules [19]. Although only less than 3% of the human genome represent protein-coding genes, most of the genome is transcribed, leading to the production of different types of RNA [21]. A more recent assessment of transcripts has revealed a variety of RNA types with different sizes and shapes that do not encode proteins but have important regulatory functions, revealing further complexity to gene expression [22]. Among these ncRNAs, microRNAs are probably the most studied class. It is estimated that they regulate over 60% of mRNAs in humans [23] by inhibiting their translation into proteins. Furthermore, a single microRNA can target several mRNAs, and each mRNA can be targeted by multiple microRNAs [24]. They have also received much attention because, unlike most RNAs, they are stable in the blood and their diversity and abundance may reflect distinct health states. Such characteristics make these small molecules very good biomarker candidates for clinically relevant parameters. Besides microRNAs, there are other types of ncRNA, including circular RNAs (also stable in the blood) and the heterogeneous class of long non-coding RNAs (lncRNAs), playing diverse functions in the cell, possibly as cell-to-cell communicators and with further potential as biomarkers for CVD [18,25].

Most of the available information about the transcriptome in CVD patients comes from studies in non-cardiac tissues and cells, such as blood and blood cells, mainly due to their accessibility [26]. Nonetheless, particularly ncRNAs have been associated with nearly all cardiovascular processes, from normal heart development to stress response in adults, regulating heart hypertrophy, contractility, fibrosis, apoptosis, and gene expression. They also modulate gene expression and cell fate in vascular endothelial and mural (smooth muscle cells and pericytes) cells, playing a role in vascular biology [27,28]. Still, there is much to explore and learn about the biological mechanisms of ncRNAs contributing to homeostasis and CVD, a task that may be complicated partly due to their diverse mechanisms of action [25]. This hampers the exploitation of the full potential of RNA-based therapeutics. Since ncRNAs are important regulators of pathophysiological processes in the cardiovascular system and are present in the blood in protected forms that prevent their degradation, they have potential to be used as diagnostic and prognostic markers and as therapeutic targets [18,25,29].

High-throughput sequencing technologies combined with bioinformatics and computational biology have recently helped expand the field of transcriptomics [30]. Three main techniques are diffusely employed to assess RNA expression: RNA sequencing (RNA-seq), microarrays, and quantitative real-time polymerase chain reaction (qPCR). The first is used to obtain a complete profile of RNA in a sample, while the others are used to detect previously characterized RNAs [31]. All techniques have been widely used to enlighten our understanding of the transcriptome. However, the field is still in its infancy and faces many challenges. First is the complexity of the whole transcriptome due to its various players, their interactions, and different mechanisms of action. These relationships and functions are still to be determined. Another issue is the lack of consensus on all steps of the process on how to best perform and interpret experiments of CVD transcriptomic studies. Comparison of results and replication of experiments are imperative to advance in gene expression studies. For example, the quality of RNA is crucial to obtain reliable results [26], especially because RNA profiles can be easily disturbed by sample collection and processing [32]. For microRNA studies, in particular, different sample types (e.g., serum, plasma, blood collection in heparin) and measurement platforms can affect the microRNA quantification results [33]. Moreover, there is an urgent need to develop internal and better external reference materials for such studies.

Thus, transcriptomics holds potential to explain fundamental biological phenomena in the developing field of ncRNAs within a CVD context and to advance medicine through the development of new diagnostic and therapeutic tools. However, for transcriptomics to fulfill the promise of improving healthcare, technical advances and a better understanding of the biological functions of ncRNA must be accomplished. This Action aims to both refine and standardize RNA analyses and reveal ncRNA molecules that might be useful to detect disease, predict its progression, and treat it.

CardioRNA believes the critical challenges regarding transcriptomics studies in CVD can be significantly mitigated by coordinating efforts from different groups through this Action, leading to progress in the quality and speed of findings in the field and, hence, in healthcare. The topics to be tackled in this Action all contribute to such advancement, and networking will allow researchers to identify and come up with innovative ways to answer difficult biological questions regarding the role of RNA in CVD.

The organisation of events and meetings during this Action will pave the way for discussions and knowledge exchange about RNA interactions that may prevent or lead to CVD, identify gaps in the current transcriptomics in CVD research, and define future directions of study, all adding to the current state-of-the-art. The Action will support the continuity of studies and projects focusing on basic and translational research. By targeting best practices on every step along a transcriptomics CVD study pipeline, the Action aims to increase comparison and reproducibility of results between different laboratories that will interest investigators beyond CardioRNA members. Among the current partners are experts on clinical biobanking and biospecimen science who can provide a valuable contribution to best practices for CVD sample collection and processing and aid in the production of guidelines to build reference materials for CVD, for instance. Furthermore, the elaboration of an inventory of available patient cohorts classified by main CVD will substantially help researchers find adequate cohorts for their studies and may facilitate collaborations.

The immediate benefits of this Action will be the advancement of scientific collaboration across Europe and the unification of transcriptomics research in CVD through the network, involving clinical, academic, and industrial partners.

## 5. Added Value of Networking

The potential applications of transcriptomics to aid manage CVD are clear; however, knowledge of its role in this context is still coming to light, especially as technologies progress and novel players and interactions emerge, together with new possibilities. A broader coordinated structure can significantly enhance existing collaborations between groups. This COST Action is well-suited for strengthening ongoing collaborations and establishing new ones by the creation of a large, interdisciplinary, and international network to jointly orchestrate and strategically plan integrated activities that will result in meaningful advancements in the field and, eventually, in healthcare. To maximize the potential of European research in the important and still developing field of transcriptomics and speed up breakthroughs, coordination of efforts is paramount to make a greater impact.

Networking through this Action will allow, not only the initiation of important discussions about the transcriptome’s role in CVD, but also allow us to search, identify, and define new directions of study and plan future projects benefiting from the expertise of members from different disciplines. Also, networking is key to deal with the current lack of consensus for best practices in transcriptomics studies for CVD, which hinders the development of RNA-based products particularly. Appropriate procedures and standards throughout the entire process to generate data from RNA are key to determine the quality of information and its later applications. Thus, an organized network with a dedicated working group (WG) focusing on best practices will facilitate the establishment, acceptance, and dissemination of standard practices due to a large number of participants and their widespread locations, extending to their networks worldwide.

## 6. Expected Impact

CardioRNA expects widespread coordination of various parties interested in the context of transcriptomics in CVD. It anticipates exchange among the members and knowledge transfer to students and young researchers. Consequently, this Action will provide the basis for significant advancements for CVD transcriptomics and translational research as well as personalized medicine, impacting society in addition to the scientific community.

In the short term, this Action will have the following scientific, technological, and socioeconomic impacts:

1. Research collaboration and joint grant applications for future national and European funding;
2. Increase the competitiveness of the network participants for European funding and the impact of resulting research;
3. Encourage translational research due to the participation of industrial partners in the network;
4. Stimulate innovative perspectives to study the transcriptome due to CardioRNA’s multidisciplinary team;
5. Provide opportunities and scientific training for ECI and students through workshops, training schools, and STSMs.

The long-term impacts of the Action include:

1. Expand fundamental knowledge about the role of the transcriptome in CVD to improve its prognostics, diagnostics, and treatment;
2. Increase the quality of data generated and reproducibility of studies of the CVD transcriptome, thus raising the quality and impact of publications;
3. Establish a leadership of both the network members and the European research axis in the proposed research field;
4. Foster personalized medicine and consequently healthcare, through outcomes of public–private collaborations.

## 7. Potential for Innovation

The main potential innovations from this Action will be the result of increasing collaboration through a coordinated initiative to optimize returns on scientific investment, filling the gap of unmet knowledge and clinical needs in CVD personalized medicine. Thus, the Action will have two main potential impacts: A scientific and technological one and a socioeconomic one. From the scientific and technological point of view, the combination of expertise from the participating research groups at the front line of transcriptomics in CVD will lead to significant breakthroughs in the knowledge level in this field and potentially to novel intellectual property for clinical applications. This, in turn, will positively impact healthcare. However, to be successful in understanding transcriptomics in CVD and fulfilling its innovation potential as biomarkers and therapeutic targets, standardization of best practices is urgent. This topic will be addressed by WG2, which will devise strategies to minimize low reproducibility of experiments, diversity of sample types, and their collection and processing. Harmonization of procedures is a prerequisite to reproducibility and further industrial developments of translational research findings. Many new therapeutic or biomarker candidates fail to be reproduced/validated due to unstandardized methodologies for blood collection, marker assessment, or even inadequate statistical analyses. CardioRNA will provide new lines of conduct to reduce the rate of failure to validate new findings from research laboratories, increasing the chances of bringing to commercial application novel RNA targets. To minimize the risk associated with harmonization of procedures, the consortium gathers experts in biobanking, biospecimen science, and bioinformatics, fields that cover topics necessary for procedures’ standardization. Discussions and exchange of experiences within these experts will reduce the risk of failure to provide new standardization guidelines to an acceptable level. Such an endeavor can only be achieved through the cooperation and coordination of research groups active in the field, which this Action will promote.

CardioRNA will also boost the success chances of personalized medicine endeavors, which require know-how from industry. The network’s industry partners are small and medium enterprise (SME) leaders in technology and experienced in developing biomarkers into diagnostic tools, discovering and designing drugs, and introducing technologies and devices into laboratories worldwide. Thus, this Action can also contribute to socioeconomic innovation by accelerating the development of products for the clinic, allowing doctors to better tailor treatment to the needs of each patient. For example, in some cases, a standard treatment is administered to every patient because there are no tools to stratify specific groups at risk, leading to increased patient burden due to unnecessary treatment and excessive healthcare costs. Additionally, RNA-based therapy potentially represents a powerful tool for personalized medicine due to their specific expression patterns associated with distinct pathologies. But for being in its early days, it faces challenges related to scientific and business aspects. CardioRNA plans to deal with these risks by building an interdisciplinary network to be able to have a systems approach to the complexity of the transcriptome and CVD and the population variability. Hence, we expect to give the CVD community successful case studies that can inspire and orient future efforts for therapy development.

## 8. Implementation Plan

**Figure 3:**
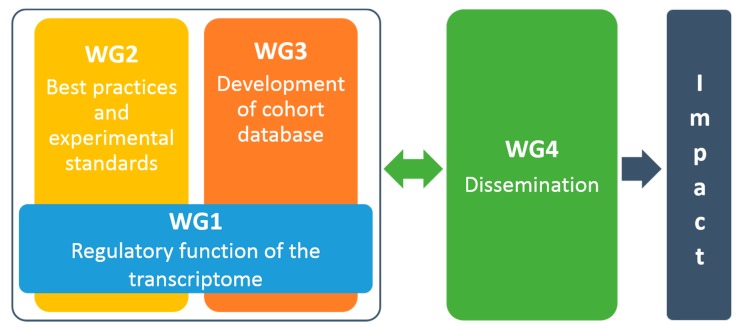
Overall interrelations between the four CardioRNA working groups (WGs) created to achieve the goals described.

CardioRNA will be organized into four interrelated working groups (WG) to optimize the accomplishment of the main proposed goals (Figure 3). Three WGs will be dedicated to developing science-related activities and meet the research coordination goals. One WG will communicate within and outside the network and support the other WGs to develop their networking activities.

### 8.1. WG1: Regulatory Function of the Transcriptome

The human transcriptome is composed of coding and non-coding transcripts. Among the non-coding transcripts, one distinguishes short ncRNAs and lncRNAs. The latter group is the largest and most diverse class of transcripts and exerts regulatory functions that ultimately control cell identity and behavior. In particular, this regulatory layer integrates developmental and environmental cues to shape the various cellular responses implicated in development and disease. Although microRNAs have been extensively studied in the context of cardiovascular disease, lncRNAs need to be systematically investigated. A better understanding of their importance as regulators of the gene programs controlling the response to stress should allow identification of new targets for improving diagnosis and treatment. WG1 will, therefore, focus on identifying novel ncRNAs and characterizing their regulatory functions in cardiovascular pathophysiology, including their interactions with protein-coding transcripts. One aspect that will be addressed is the difference of ncRNA expression according to gender. This WG will provide training in the various approaches and techniques that are used to probe and manipulate the transcriptome to study the role of ncRNAs in the cardiovascular system.

### 8.2. WG2: Best Practices and Experimental Standards

Comparison of protocols and data from different laboratories is necessary to facilitate the comprehension of results and advance knowledge in CVD transcriptomics. It also directly impacts the development of tools that can be used for diagnosis and monitoring of CVD. To compare results, generated data must meet common standards, which should be from the collection of samples to the generation and analysis of data, so that variations are minimized and results are accurate and reproducible. Sample collection, storage, and quality testing are critical, particularly when dealing with RNA due to its sensitive nature. Although guidelines exist for separate steps of sample collection, data generation, and analysis, a complete guideline comprising all steps that are specific for CVD is still lacking. WG2 will focus on the best practices for cardiovascular transcriptomic studies from the crucial issue of sample collection and processing, which directly impacts final results, to the main techniques used to study the transcriptome (qPCR, microarray, and RNA sequencing) and the statistical analysis. Protocols, technologies, and procedures will be assessed, and standard strategies will be delineated for better quality control and study comparison, tackling all the steps along the process. This Action aims to bring together in WG2 experts from relevant disciplines to discuss and come up with guidelines to generate reference materials for CVD and also for the production of internal quality control materials that can be used in CVD transcriptome analyses. 

### 8.3. WG3: Development of Cohort Inventory

The main goal of WG3 is to create an inventory of cohorts from healthy individuals and CVD patients to provide awareness of the available cohorts that can be used in research, thus, facilitating collaborations. Such an inventory, with key characteristics of the cohort (paying particular attention to gender) and information on the principal investigator, will be made available to the public through this COST Action’s website and possibly through websites from other relevant organizations and institutions (e.g., Biobanking and BioMolecular Resources Research Infrastructure Directory, International Society for Biological and Environmental Repositories). 

### 8.4. WG4: Dissemination

WG4 will be responsible for promoting communication, knowledge exchange, and bringing new members to the network. This WG will organize yearly CardioRNA network meetings and provide support for the different WGs to organize their meetings, workshops, STSMs, and training schools. It will also be responsible for providing updates on the activities of the network and disseminating the Action’s progress and initiatives to the public.

## 9. Concluding Remarks

In summary, our COST Action aims to help alleviate the global burden of CVD by addressing the following challenges: Increase the knowledge of the role played by the complex transcriptome in CVD by coordinating efforts from different groups; refine common standards and best practices that will offer the greatest potential for transcriptome studies in CVD as well as increase reproducibility between studies and advance product development; and assure continuity of studies focusing on translational research and public–private partnerships to enhance personalized medicine.

Represented by scientists with multidisciplinary backgrounds and SMEs distributed throughout Europe and beyond, this Action’s initiatives will potentially expand within the participants existing collaboration networks. Both the main subject of CardioRNA and the lack of coordination of efforts to deal with it are widespread, making cooperation beneficial.

## Figures and Tables

**Table 1:** Research objectives of CardioRNA.

Specific	Measurable	Achievable	Relevant	Timely
Further the understanding of the transcriptome’s role in CVD by establishing a network	Number of institutions and investigators joining the network	Active and increasing collaborative research activity in the field as seen in the biomedical literature	Increased knowledge on the subject has noteworthy implications for the healthcare of patients with CVD	Expansion of the network will be continuous (month 1–48)
Foster collaborative initiatives in CVD transcriptomics	Number of Action outputs such as projects submitted for funding and scientific publications	Collaborations will allow optimization and standardization of protocols, thus helping to deal with the complexity of studies	Join expertise to aid European research groups improve and consolidate their research capacity and leadership in this field	Project application to different funding bodies per year (month 12–48)
Develop improved guidelines for best practices and experimental standards that offer the greatest potential for cardiovascular transcriptomics studies	Production of specific documents (peer-reviewed publications and others)	Different guidelines and techniques are successfully used in this field of research	Increasing standardization will facilitate comparison and reproducibility of results, allowing faster interpretation of results and development of new tools to the clinic	Publish documents on (1) best practices on collection and processing of biological material (month 24); (2) experimental standards for RNA analysis in CVD (month 40)
Stimulate development and optimization of RNA-based products for prognostic, diagnostic and therapy for CVD management	Number of projects that target translational research and personalized medicine	Partnerships from the network will facilitate study designs on the translation of research knowledge into medicinal products	Regards healthcare improvement of patients with CVD	Projects on clinical products development (month 48)
